# Acute Respiratory Infections in Ghanaian Children: Epidemiology, Antimicrobial Resistance, and Prevention Strategies

**DOI:** 10.3390/pathogens15030285

**Published:** 2026-03-06

**Authors:** Sabastine Eugene Arthur, Jessica Eyeson, Aaron Appiah Kubi, Faustina Amarteley Amartey, Raymond Matey, James Odame Aboagye, George Boateng Kyei

**Affiliations:** 1 Medical and Scientific Research Centre, University of Ghana Medical Centre, Legon, Accra GA-337-6980, Ghana; sarthur@ugmc.ug.edu.gh (S.E.A.); jesseyeson15@gmail.com (J.E.); aaronkubi18@gmail.com (A.A.K.); amarteyfaustina123@gmail.com (F.A.A.); rmatey@ugmc.ug.edu.gh (R.M.); 2 Noguchi Memorial Institute for Medical Research, University of Ghana, Legon, Accra GA-337-3045, Ghana; 3 Departments of Medicine and Molecular Microbiology, Washington University School of Medicine, St. Louis, MO 63110, USA

**Keywords:** pediatric acute respiratory infections, children under five, Ghana, COVID-19 pandemic, post-pandemic surveillance, antimicrobial resistance, vaccination/immunization

## Abstract

Acute respiratory infections (ARIs) remain a common cause of morbidity and mortality in children, especially in sub-Saharan Africa, where countries such as Ghana are severely affected. This review presents recent data on ARI etiology, clinical burden, and antimicrobial resistance (AMR) from Ghana, spanning the pre-COVID-19 era (2010–2019) to the post-pandemic period (2020–2025). Before the COVID-19 pandemic, viral infections, such as respiratory syncytial virus (RSV), rhinoviruses, and influenza viruses, were the major contributors, along with established bacterial pathogens such as *Streptococcus pneumoniae* and *Haemophilus influenzae*. Social determinants, including undernutrition and indoor air pollution, also influenced these infections. In the COVID era, we have seen dramatic shifts in pathogen seasonality, the scaling of oxygen delivery systems, and the implementation of genomic surveillance for SARS-CoV-2, as well as new features such as maternal RSV vaccination and monoclonal antibody therapy. Despite its successes in vaccination coverage and health system strengthening, some challenges remain, including fluctuations in implementation and surveillance issues. The simultaneous challenges of pneumonia and hygiene will require integrated, coordinated, multisectoral responses that incorporate surveillance with antibiotic stewardship, sustainable oxygen systems, and interventions for nutrition and environmental health. The review also highlights research priorities and makes policy recommendations well aligned to support national ARI control efforts aimed at reducing child mortality due to ARI and achieving Sustainable Development Goals targets on child health.

## 1. Introduction

Acute respiratory infections (ARIs) remain a leading cause of morbidity and mortality among children under five years globally, particularly in low- and middle-income countries (LMICs), where inequities in healthcare access, nutrition, and living conditions exacerbate vulnerability [1]. Despite progress in immunization coverage and child survival, ARIs remain a substantial public health concern in Ghana.

According to the 2022 Ghana Demographic and Health Survey [2], approximately 2% of Ghanaian children aged 6–59 months experienced symptoms consistent with ARI (caregiver-reported cough plus rapid or difficult breathing) in the prior two weeks. In further analysis, Klu, Alhassan, and Dansu [3] demonstrate that this burden is higher in rural communities, among poorer households, and among mothers with lower educational attainment. Many studies employ broader symptom-based definitions of ARI, typically relying on caregiver reports of cough with fast or difficult breathing, rather than the full WHO/IMCI clinical criteria that distinguish between non-severe and severe pneumonia [4,5,6]. These broader definitions tend to yield higher prevalence estimates. For example, the Ghana Demographic and Health Survey (DHS) excludes cases of nasal congestion alone in its ARI definition [2,7]. Similar definitions are used across low- and middle-income settings [3,8]. However, not all studies clearly specify how they operationalize symptoms, which makes comparisons difficult [9]. Socioeconomic and environmental factors, such as indoor air pollution, poor sanitation, overcrowding, and undernutrition, remain key determinants of both symptom risk and progression to more severe disease [3,10]. Regional analyses across sub-Saharan Africa consistently reinforce that modifiable household and community factors, particularly maternal education, significantly influence ARI risk, suggesting that patterns observed in Ghana (e.g., the impacts of sanitation and indoor pollution) also apply elsewhere [11,12].

Before the COVID-19 pandemic, Ghana’s pediatric ARI landscape was dominated by viral pathogens, particularly respiratory syncytial virus (RSV), influenza viruses, and parainfluenza, alongside bacterial infections caused by *S. pneumoniae* and *H. influenzae* type b [13,14]. The introduction of the pneumococcal conjugate vaccine (PCV) and the *H. influenzae* type b (Hib) vaccine has markedly reduced invasive bacterial disease; however, incomplete vaccine coverage and the persistence of serotype replacement have constrained the full impact of these vaccines [15,16]. Additional risk modifiers such as malaria co-infection and variable maternal literacy have been shown to influence ARI risk and clinical outcomes in Ghanaian children [3,17].

The COVID-19 pandemic (2020–2022) significantly disrupted routine child health services and altered patterns of pathogen circulation. Public health measures, such as masking, school closures, and reduced mobility, contributed to altered seasonality and reduced transmission of several viral pathogens. However, health service disruptions, changes in care-seeking behavior, and increased empirical antibiotic use introduced new challenges [18,19]. Notably, Ghana’s pandemic response accelerated investments in molecular diagnostics and genomic sequencing, which have since been leveraged for broader surveillance of respiratory pathogens. The integration of SARS-CoV-2 surveillance into the existing influenza surveillance network exemplifies this approach, enhancing the country’s capacity to monitor and respond to respiratory pathogens [18].

Ghana now stands at a critical juncture in its efforts to strengthen its pediatric ARI prevention and control framework. The expected rollout of maternal RSV vaccination and monoclonal antibody prophylaxis offers new opportunities to reduce severe infant pneumonia [20,21,22]. However, growing antimicrobial resistance (AMR), uneven access to diagnostics, and persistent social and environmental inequities threaten to reduce the impact of current and future advances in prevention and care [18,19].

This narrative review synthesizes evidence on Ghana’s pediatric ARI burden in the decade before and the 5 years after the COVID-19 pandemic, emphasizing pathogen ecology, antimicrobial stewardship, diagnostic and oxygen system capacity, and emerging immunization strategies. By contrasting these time periods, it identifies progress, enduring gaps, and policy priorities toward achieving Sustainable Development Goal 3.2, adopted by the United Nations General Assembly in 2015 as part of the 2030 Agenda for Sustainable Development, which aims to end preventable deaths among children under five, years of age, with targets of reducing neonatal mortality to at least as low as 12 per 1000 live births and under-five mortality to at least as low as 25 per 1000 live births [23].

## 2. Methods

This study is a narrative review that synthesizes published and gray literature on pediatric acute respiratory infections (ARIs) in Ghana, spanning both the pre-COVID-19 and post-COVID-19 periods. For the review, we employed a transparent evidence synthesis approach adapted from the PRISMA-ScR framework for assessing reviews, ensuring clarity in reporting study selection, data extraction, and thematic synthesis. No quantitative meta-analysis was conducted.

The review encompasses literature published between 2010 and 2025, divided into two analytical phases: the pre-COVID-19 era (2010–2019), which reflects baseline epidemiology and interventions, and the post-COVID-19 era (2020–2025), capturing pandemic-related changes in pathogen circulation, health service delivery, and public health responses.

A comprehensive search was conducted across the PubMed, Scopus, Web of Science, and Google Scholar databases, complemented by gray literature from key national and international organizations, including the Ghana Health Service, the Ministry of Health, the World Health Organization, and UNICEF. Search terms were combined to include keywords related to respiratory infections, children, Ghana, and pandemic or post-pandemic dynamics, alongside relevant pathogens and health system terms, to maximize the retrieval of pertinent studies. Additional citation cross-checking was conducted to identify relevant literature that was missed in the initial searches.

Studies were included if they reported quantitative or qualitative data on the burden, etiology, diagnosis, management, or prevention of ARIs among Ghanaian children under five years old, and if the findings were relevant to health system performance or child health policy. Only English-language publications from 2010 through 2025 were considered. National, regional, and facility-based studies were eligible for inclusion. Exclusion criteria included case reports, studies lacking clear data sources or methodology, non-Ghanaian populations, and non-English publications.

Screening and data extraction were conducted independently by two reviewers, with discrepancies resolved by consensus to minimize bias. The extracted data included the study design, population characteristics, key findings, and implications for ARI prevention and control in Ghana.

Extracted data were synthesized thematically across six domains: pathogen ecology and epidemiology, surveillance and diagnostics, antimicrobial resistance and stewardship, oxygen therapy and health system capacity, vaccination and preventive programs, and policy and social determinants. Narrative synthesis integrated evidence from peer-reviewed and policy literature, with findings cross-validated against national datasets such as the 2022 Ghana Demographic and Health Survey and Ghana Health Service surveillance reports to ensure contextual accuracy.

No formal risk-of-bias or quality appraisal was performed, given the narrative nature of the synthesis; however, the heterogeneity and methodological limitations of the included studies were critically considered during the interpretation of the literature.

As this review exclusively utilized publicly available data and literature, ethical approval was not required.

This narrative review has several methodological limitations. The included studies vary widely in design, case definitions, diagnostic methods, and surveillance approaches, ranging from hospital-based studies to national surveys and sentinel surveillance reports. Such heterogeneity limits direct quantitative comparisons across time periods and settings and necessitates cautious interpretation of trends. In addition, reliance on published and gray literature may introduce publication and reporting biases, and the absence of a formal risk-of-bias assessment reflects the narrative rather than systematic nature of the synthesis.

## 3. Pre-COVID-19 Era (2010–2019)

In the decade preceding the COVID-19 pandemic, acute respiratory infections (ARIs) remained a leading cause of pediatric morbidity and mortality in Ghana, despite significant national progress in child health indicators [2]. Pneumonia accounted for a substantial proportion of hospital admissions and under-five deaths, with incidence estimates exceeding 300 cases per 1000 child-years in some regions [6,24]. These figures underscore the disproportionate burden in sub-Saharan Africa, where gaps in preventive and curative care magnify the impact of respiratory infections [25].

Viral agents dominated the etiological spectrum. Respiratory syncytial virus (RSV) remained the single most important pathogen, accounting for approximately 18–32% of viral detections among hospitalized children with severe lower respiratory tract infections in Ghana [26,27]. Seasonal peaks occurred during the rainy season, reflecting the influence of climatic factors on viral propagation [28,29]. In addition to RSV, concurrent circulation of influenza (6–12% of detections), parainfluenza (6–7%), and rhinoviruses (11–15%) contributed substantially to the overall ARI burden, complicating outbreak prediction and response [24,26].

Table 1 provides a concise summary of the prevalence rates and key pathogens identified during the three periods discussed in this review. The data highlights significant shifts in the etiological landscape of pediatric acute respiratory infections following the onset of the COVID-19 pandemic, as well as the impact of public health interventions and environmental factors on pathogen circulation.

Bacterial pneumonia, primarily caused by Streptococcus pneumoniae and Haemophilus influenzae type b, remained an important cause of severe disease and mortality [30,31]. Nasopharyngeal carriage of *S. pneumoniae* was high in study populations: Dayie et al. [31] reported an overall carriage prevalence of 54% (95% CI 49–59%) among children < 5 years in Accra five years after 13-Valent Pneumococcal Conjugate Vaccine (PCV13) introduction, with ~20% carrying PCV-13 vaccine serotypes and ~37% carrying non-PCV-13 serotypes. In HIV-infected children, Donkor et al. found a carriage prevalence of 27.1% (95% CI 19.1–35.1%), with a PCV-13 serotype coverage of 41.5% among isolates [30,31]. The rollout of PCV13 in 2012 and Hib vaccination significantly reduced invasive infections; however, the substantial residual carriage and the predominance of non-vaccine serotypes, together with high inpatient antibiotic use reported in tertiary facilities, underscore the need for ongoing surveillance and adaptive immunization and antimicrobial-stewardship strategies [14,30,31,34].

Clinically, hypoxaemia consistently emerged as a major predictor of poor outcomes, underscoring the importance of oxygen therapy for survival [35,36]. However, oxygen availability remained limited, particularly in lower-level facilities, and constrained diagnostic capacity hindered early identification and management [37]. Comorbidities, such as malnutrition and HIV infection, further increased vulnerability, underscoring the complexity of ARI care in high-risk subpopulations [38].

Environmental and socio-demographic determinants, including household air pollution from biomass fuel, poor sanitation, low maternal education, and socioeconomic disadvantage, were key drivers of ARI risk and severity, as shown in multi-country surveys across sub-Saharan Africa [11]. These findings demonstrate the intersection of socio-environmental factors with infectious disease dynamics, highlighting the need for integrated interventions.

Antibiotic overuse and misuse were widely documented during this period. Point-prevalence data showed that about 51 to 52% of hospitalized patients were receiving at least one antibiotic, and more than 80% of these prescriptions were based on empiric treatment rather than culture results. In primary care settings, inappropriate use was also common. Prah et al. (2017) reported that 67.2% of all outpatient consultations resulted in an antibiotic prescription, many for conditions that were not likely to be bacterial [39]. These patterns, combined with limited microbiologic diagnostic capacity and inadequate clinical decision support, contributed to rising antimicrobial resistance among important pathogens, including multidrug-resistant pneumococci and Gram-negative bacteria [40,41,42]. Although Ghana’s National Action Plan for AMR (2017–2021) introduced stewardship interventions, implementation during this period was inconsistent.

In summary, the pre-COVID-19 era in Ghana was marked by a substantial pediatric ARI burden driven by viral and bacterial pathogens, compounded by socio-environmental vulnerabilities and health-system constraints. While vaccine deployment and limited access to oxygen represent pockets of progress, persistent gaps in surveillance, diagnostics, and antimicrobial stewardship underscore areas that need urgent investment.

## 4. Bridging the Pre- and Post-Pandemic Dynamics

Despite the continued prominence of viral and bacterial pathogens, the COVID-19 pandemic triggered substantial disruptions and innovations. Seasonal transmission patterns shifted, healthcare-seeking behaviors changed, and preventive services faced interruptions [34,43,44]. Concurrently, the pandemic accelerated systemic improvements, including the expansion of oxygen infrastructure, enhancement of molecular diagnostic capacity, and repurposing of genomic surveillance platforms to support multi-pathogen monitoring [18]. Persistent socio-environmental and educational disparities continue to challenge ARI control, but novel preventive tools, including maternal RSV vaccines and monoclonal antibodies, offer promising opportunities to reduce morbidity and mortality [11,20,21].

## 5. Post-COVID-19 Era (2020–2025)

The COVID-19 pandemic profoundly reshaped ARI epidemiology, clinical management, and health system responses in Ghana. Lockdowns, masking, and school closures disrupted immunization programs, disease surveillance, and healthcare delivery, leading to altered pathogen circulation and delayed RSV seasonality in 2022–2023 [18]. These disruptions raised concerns about the potential resurgence of vaccine-preventable bacterial pneumonias.

In response, Ghana invested heavily in expanding oxygen-delivery infrastructure across tertiary and district hospitals and widely adopted pulse oximetry, thereby improving hypoxemia management [36,45]. Despite these advances, empirical antibiotic use increased due to clinical uncertainty and overlapping COVID-19 symptoms, intensifying AMR risks. Analyses of pharmacy and hospital data have documented heightened broad-spectrum antibiotic consumption, prompting the reinforcement of Ghana’s AMR action plan with One Health frameworks and the expansion of laboratory capacity [41,46,47].

Innovative preventive tools, such as maternal RSV vaccination and long-acting monoclonal antibodies (nirsevimab), endorsed by the WHO in 2024, offer promising strategies to reduce severe infant pneumonia further [20,21,48]. Ghana is poised for an equity-focused rollout within its Expanded Program on Immunization, requiring readiness assessments and robust implementation strategies [49,50,51].

Genomic surveillance platforms, initially established for SARS-CoV-2, have been repurposed for multi-pathogen respiratory surveillance, strengthening early detection, public health response, and epidemic preparedness [18].

Persistent inequities in healthcare access and infrastructure, interruptions in preventive services, and ongoing socio-environmental vulnerabilities, including modifiable household risk factors and educational disparities, continue to challenge optimal ARI management [11].

Taken together, Ghana’s post-pandemic pediatric ARI landscape is characterized by dynamic pathogen patterns, adaptive health system responses, and emerging preventive opportunities. Consolidating gains in oxygen therapy, diagnostics, surveillance, and immunization, while addressing inequities and modifiable risk factors, is essential to sustain progress and achieve Sustainable Development Goal 3.2: ending preventable deaths among children under five.

The evolving pediatric ARI landscape in Ghana, shaped by shifts in pathogen patterns, expanded oxygen infrastructure, strengthened surveillance, and emerging preventive innovations, highlights both progress and persistent inequities. Table 2 summarizes key contrasts between the pre- and post-COVID-19 eras, integrating evidence across pathogen dynamics, surveillance capacity, antimicrobial resistance, oxygen access, and policy frameworks.

Direct comparative estimates of pediatric acute respiratory infection (ARI)–specific morbidity and mortality between the pre- and post-COVID-19 periods in Ghana remain limited, reflecting heterogeneity in surveillance systems, case definitions, and reporting completeness across studies [9,25]. Nevertheless, available evidence provides important insights into overall trends.

Globally, non-pharmaceutical interventions implemented during the COVID-19 pandemic were associated with marked reductions in the circulation of common respiratory viruses, including influenza and respiratory syncytial virus, with corresponding short-term declines in ARI-related morbidity and mortality [25,55]. In many settings, this was followed by a post-pandemic resurgence of respiratory pathogens, in some cases exceeding pre-pandemic incidence levels [19,55].

In Ghana, pandemic-related restrictions and changes in healthcare-seeking behavior were associated with reduced ARI-related hospital presentations during the height of COVID-19 control measures, followed by increased ARI burden as restrictions eased and routine transmission patterns resumed [18,19]. While overall under-five mortality in Ghana has continued a gradual long-term decline driven by improvements in vaccination coverage and healthcare access, acute respiratory infections remain among the leading causes of childhood morbidity and mortality [56].

The post-pandemic period is therefore characterized by altered pathogen dynamics, indirect health system effects, and persistent structural vulnerabilities, underscoring the importance of sustained surveillance, strengthened preventive programs, and integrated child health interventions [25].

## 6. Synthesis and Implications

Synthesizing the available evidence, this review highlights the evolving landscape of pediatric acute respiratory infections (ARIs) in Ghana, spanning both pre- and post-COVID-19 eras. Pre-pandemic, the ARI burden was driven by a complex interplay of viral and bacterial pathogens, persistent socio-environmental vulnerabilities, and health system constraints, including limited oxygen and diagnostic capacity and widespread antimicrobial misuse [11,35,38,52]. Post-pandemic, systemic disruptions shifted pathogen circulation and healthcare delivery but concurrently catalyzed health system strengthening, notably through expanded oxygen infrastructure, enhanced molecular diagnostics, and repurposed genomic surveillance platforms [18]

Key implications for policy and practice emerge from these dynamics. Based on the synthesized evidence, sustained investment in oxygen and diagnostic infrastructure is critical to reducing mortality from severe ARIs, particularly in rural and underserved regions [36,45]. Equally important is strengthening antimicrobial stewardship to counter the rising antimicrobial resistance (AMR), especially in settings with high empirical antibiotic use [41,47,57]. The introduction of maternal RSV vaccines and long-acting monoclonal antibodies presents a novel preventive strategy that requires an equity-focused rollout and seamless integration into routine immunization programs [20,21,48].

The social determinants of health remain central drivers of ARI risk. Modifiable risk factors, including household air pollution, low maternal education, and socio-economic disadvantage, persist across populations [3,11]. Addressing these determinants through coordinated, multisectoral interventions that span sanitation, nutrition, and maternal education will be vital to amplify the benefits of biomedical advances and reduce disparities.

Research priorities are clear and urgent. Ongoing monitoring of pathogen ecology, rigorous evaluation of novel immunization strategies, and assessment of the long-term impacts of COVID-19-related service disruptions are essential [18,19]. Implementation science approaches can guide the tailoring of context-specific interventions and optimize resource allocation to achieve the maximum health impact.

In summary, Ghana’s pediatric ARI burden reflects the complex intersection of pathogen ecology, health system capacity, and socio-environmental realities. Policy and programmatic initiatives must prioritize integrated, multisectoral strategies that consolidate post-pandemic gains while systematically addressing persistent disparities. Doing so is critical to advancing toward Sustainable Development Goal 3.2 and significantly reducing preventable childhood mortality.

## 7. Policy Implications and Future Directions

Translating the synthesized evidence into action, this section outlines policy implications and future directions for strengthening pediatric ARI prevention and control in Ghana. Sustained progress in reducing pediatric ARIs in Ghana demands an integrated approach that consolidates lessons from advancing surveillance, antimicrobial stewardship, and vaccination programs. Strengthening surveillance systems is paramount, leveraging advances driven by COVID-19 to build resilient, real-time pathogen monitoring networks. The expansion of molecular diagnostic capacity and scaling up of integrated disease surveillance and response (IDSR) frameworks at the district level can help close enduring gaps, particularly in rural and underserved areas [33]. Investment in mobile health technologies and community engagement will further facilitate timely data collection, enhance public health messaging, and promote adherence to healthcare-seeking behavior [58].

Antimicrobial resistance remains a formidable threat, exacerbated by the pandemic-era use of empirical and broad-spectrum antibiotics. Addressing AMR requires the sustained strengthening of stewardship programs, embedded in a One Health framework, with multisector collaboration across human, animal, and environmental health sectors, and enhanced laboratory networks for precise diagnostics and AMR surveillance [47]. Building the capacity for rational antibiotic use among providers and caregivers through education, clear guidelines, and regulatory enforcement is critical to preserving therapeutic efficacy.

Vaccination programs must sustainably expand with an emphasis on universal access and equity. The integration of maternal RSV vaccines and monoclonal antibodies into the national immunization schedule has the potential to transform the prevention of severe infant pneumonia. Their success depends on robust delivery frameworks ensuring timely and equitable coverage [20,21,48]. Pneumococcal and Hib vaccination programs require ongoing optimization supported by surveillance of serotype replacement and vaccine effectiveness.

Sustaining and expanding oxygen and diagnostic capacity beyond pandemic-scale-up is essential for improved clinical outcomes. Reliable oxygen supply chains, widespread availability of oxygen concentrators and pulse oximeters at primary and secondary levels, and continuous staff training with quality assurance underpin effective hypoxaemia management [45]. The deployment of point-of-care diagnostics for common respiratory pathogens can facilitate targeted treatment and further strengthen stewardship efforts.

Addressing the social and environmental determinants of ARIs must remain a core component of strategy. Interventions to decrease indoor air pollution via cleaner cooking technologies, improve nutrition and maternal health, and enhance sanitation and living conditions are vital to reducing ARI incidence and progression [3,38]. Tailored community-based initiatives that are sensitive to socioeconomic vulnerabilities are crucial for narrowing urban–rural health disparities and encouraging timely healthcare-seeking behavior.

Finally, closing critical research gaps requires focused efforts. This includes investigating the long-term impacts of COVID-19 on ARI epidemiology, evaluating newly introduced vaccines and monoclonal antibodies in real-world settings, and mapping context-specific patterns of antimicrobial use. Implementation science research is crucial for optimizing the integration of evolving diagnostics, oxygen therapy, and preventive interventions into routine care.

Ultimately, multisectoral collaboration engaging health, environment, education, and social protection sectors is fundamental. Policymakers must champion comprehensive, coordinated strategies combining surveillance, prevention, treatment, social interventions, and capacity building to achieve sustained reductions in Ghana’s pediatric ARI burden and meet global child-health targets.

## 8. Conclusions

Pediatric acute respiratory infections (ARIs) in Ghana continue to pose a significant health burden, influenced by evolving pathogen dynamics, antimicrobial resistance, and inequities in healthcare access [41,47,59]. The COVID-19 pandemic accelerated health system adaptations, including the expansion of genomic surveillance, improvements in oxygen delivery, and enhanced stewardship programs [18,36,45].

Sustaining these gains requires integrated approaches that combine surveillance, rational antibiotic use, equitable vaccination strategies, including maternal RSV immunization and monoclonal antibodies, and strengthened diagnostic and oxygen capacity [20,21,48]. By addressing social, environmental, and system-level determinants, Ghana can further reduce ARI morbidity and mortality among children and progress toward global child-health targets.

## Figures and Tables

**Table 1:** Prevalence and key pathogens associated with pediatric acute respiratory infections in Ghana across pre-, during, and post-COVID-19 periods.

Period	Prevalence	Key Pathogens	References
Pre-COVID-19	RSV 18–32%; Influenza 6–12%; Parainfluenza 6–7%; Rhinovirus 11–15%; Streptococcus pneumoniae carriage 54% (children < 5)	RSV, Influenza, Parainfluenza, Rhinovirus, *S. pneumoniae,* Hib	Kwofie et al., 2012 [26]; Wilson et al., 2018 [27]; Bonney et al., 2012 [28]; Hogan et al., 2017 [29]; Donkor et al., 2017 [30]; Dayie 2019 [31]
During COVID-19	RSV reduced over 80%; Influenza reduced by 60–80%	SARS-CoV-2, RSV (reduced), Influenza (reduced)	Duedu 2024 [32]; Asante et al., 2024 [33]
Post-COVID-19	RSV increased to 2–3× pandemic levels; Influenza returned to 6–12%	RSV (rebound), Influenza	Adu-Gyamfi et al. [19]; Asante 2023 [18]

**Table 2:** Comparative summary of pediatric acute respiratory infection (ARI) trends in Ghana before and after the COVID-19 pandemic.

Domain	Pre-COVID-19 Era (2010–2019)	Post-COVID-19 Era (2020–2025)
Pathogen trends	Predictable seasonal viral circulation with RSV dominance; persistent pneumococcal and Hib burden [26,31].	Disrupted seasonality with delayed RSV peaks; integrated genomic surveillance for SARS-CoV-2 and other respiratory viruses [18].
Surveillance capacity	Primarily hospital-based, limited molecular diagnostics, and sparse community data [24,28].	Enhanced multiplex genomic surveillance and real-time monitoring platforms [18].
Antimicrobial use and resistance	Widespread empiric antibiotic use leading to increasing pneumococcal and Gram-negative resistance [14,40,52].	Increased broad-spectrum antibiotic use during COVID-19; strengthened AMR stewardship and One Health monitoring [11,47].
Oxygen access and case management	Limited oxygen availability mainly at tertiary hospitals; inconsistent pulse oximetry [35,37].	Major infrastructure expansion and widespread oximetry use, though regional inequities persist [45,53].
Policy and programs	Introduction of PCV and Hib vaccines; early National AMR Plan implementation; uneven integrated community case management [31].	WHO-endorsed maternal RSV vaccines and monoclonal antibodies (nirsevimab); equity-focused pandemic recovery and resilience plans [20,48,54].

Abbreviations: ARI: Acute Respiratory Infection; RSV: Respiratory Syncytial Virus; Hib: H. influenzae type b; AMR: Antimicrobial Resistance; PCV: Pneumococcal Conjugate Vaccine; WHO: World Health Organization.

## Data Availability

No new data were created or analyzed in this study. Data sharing is not applicable to this article.

## References

[1] Young M., Wolfheim C., Marsh D.R., Hammamy D. (2012). World Health Organization/United Nations Children’s Fund Joint Statement on Integrated Community Case Management: An Equity-Focused Strategy to Improve Access to Essential Treatment Services for Children. Am. J. Trop. Med. Hyg..

[2] Ghana—Demographic and Health Survey 2022. https://microdata.worldbank.org/index.php/catalog/6122.

[3] Klu D., Alhassan A., Dansu C.A. (2025). Acute respiratory infections and its associated risk factors among children aged 6–59 months in Ghana: A multinomial regression analysis of the 2022 demographic and health survey. Front. Public Health.

[4] Patwari A.K., Raina N. (2002). Integrated Management of Childhood Illness (IMCI): A robust strategy. Indian J. Pediatr..

[5] WHO/UNICEF IMCI Adaptation Guide 2c. https://cdn.who.int/media/docs/default-source/mca-documents/child/imci-integrated-management-of-childhood-illness/adaptation-guide/imci_adaptation_guide_2c.pdf?sfvrsn=a483cecf_5.

[6] WHO Pneumonia in Children. https://www.who.int/news-room/fact-sheets/detail/pneumonia.

[7] DHS Symptoms of Acute Respiratory Infection (ARI) and Careseeking. https://dhsprogram.com/Data/Guide-to-DHS-Statistics/Symptoms_of_Acute_Respiratory_Infection_ARI_and_careseeking.htm.

[8] Varghese J.S., Muhammad T. (2023). Prevalence, potential determinants, and treatment-seeking behavior of acute respiratory infection among children under age five in India: Findings from the National Family Health Survey, 2019–2021. BMC Pulm. Med..

[9] Apanga P.A., Kumbeni M.T. (2021). Factors associated with diarrhoea and acute respiratory infection in children under-5 years old in Ghana: An analysis of a national cross-sectional survey. BMC Pediatr..

[10] Prüss-Üstün A., Wolf J., Corvalán C., Bos R., Neira M. (2016). Preventing Disease Through Healthy Environments: A Global Assessment of the Burden of Disease from Environmental Risks.

[11] Ahmed S.K., Hussein S., Qurbani K., Ibrahim R.H., Fareeq A., Mahmood K.A., Mohamed M.G. (2024). Antimicrobial resistance: Impacts, challenges, and future prospects. J. Med. Surg. Public Health.

[12] Tessema G.A., Kinfu Y., Dachew B.A., Tesema A.G., Assefa Y., Alene K.A., Aregay A.F., Ayalew M.B., Bezabhe W.M., Bali A.G. (2021). The COVID-19 pandemic and healthcare systems in Africa: A scoping review of preparedness, impact and response. BMJ Glob. Health.

[13] Dayie N.T.K.D., Tetteh-Ocloo G., Labi A.-K., Olayemi E., Slotved H.-C., Lartey M., Donkor E. (2018). Pneumococcal carriage among sickle cell disease patients in Accra, Ghana: Risk factors, serotypes and antibiotic resistance. PLoS ONE.

[14] Labi A.-K., Obeng-Nkrumah N., Sunkwa-Mills G., Bediako-Bowan A., Akufo C., Bjerrum S., Owusu E., Enweronu-Laryea C., Opintan J.A., Kurtzhals J.A.L. (2018). Antibiotic prescribing in paediatric inpatients in Ghana: A multi-centre point prevalence survey. BMC Pediatr..

[15] Kobayashi M., Abdul-Karim A., Milucky J.L., Zakariah A., Leidner A.J., Asiedu-Bekoe F., Opare D., Eleeza J.B., Ofosu W., Walker C. (2021). Estimating the economic burden of pneumococcal meningitis and pneumonia in northern Ghana in the African meningitis belt post-PCV13 introduction. Vaccine.

[16] Ibrahim A.-M., Owusu R., Nonvignon J. (2024). Sustainability of pneumococcal conjugate vaccination in Ghana: A cost-effectiveness analysis in the context of donor transition. Front. Public Health.

[17] Orish V.N., Ansong J.Y., Anagi I.B., Onyeabor O.S., Sanyaolu A.O., Iriemenam N.C. (2015). Malaria and associated co-morbidity in children admitted with fever manifestation in Western Ghana: A retrospective study. J. Infect. Dev. Ctries..

[18] Asante I.A., Hsu S.N., Boatemaa L., Kwasah L., Adusei-Poku M., Odoom J.K., Awuku-Larbi Y., Foulkes B.H., Oliver-Commey J., Asiedu E.K. (2023). Repurposing an integrated national influenza platform for genomic surveillance of SARS-CoV-2 in Ghana: A molecular epidemiological analysis. Lancet Glob. Health.

[19] Adu-Gyamfi C., Asamoah J.A., Frimpong J.O., Larbi R., Ansah R.O., Aryeetey S.N.A., Gorman R., Acheampong H.K., Nyarko-Afriyie E., Hayford M. (2025). Prevalence of common respiratory viruses in children: Insights from post-pandemic surveillance. BMC Infect. Dis..

[20] Hammitt L.L., Dagan R., Yuan Y., Cots M.B., Bosheva M., Madhi S.A., Muller W.J., Zar H.J., Brooks D., Grenham A. (2022). Nirsevimab for Prevention of RSV in Healthy Late-Preterm and Term Infants. N. Engl. J. Med..

[21] Kampmann B., Madhi S.A., Munjal I., Simões E.A.F., Pahud B.A., Llapur C., Baker J., Marc G.P., Radley D., Shittu E. (2023). Bivalent Prefusion F Vaccine in Pregnancy to Prevent RSV Illness in Infants. N. Engl. J. Med..

[22] WHO Recommends Maternal Vaccine and Antibody Shot to Prevent RSV in Infants|Reuters. https://www.reuters.com/business/healthcare-pharmaceuticals/who-recommends-maternal-shot-antibody-therapy-prevent-rsv-infants-2024-10-01/.

[23] United Nations Transforming Our World: The 2030 Agenda for Sustainable Development|Department of Economic and Social Affairs. https://sdgs.un.org/2030agenda.

[24] Ntiri M.P., Duque J., McMorrow M.L., Frimpong J.A., Parbie P., Badji E., Nzussouo N.T., Benson E.-M., Adjabeng M., Dueger E. (2016). Incidence of medically attended influenza among residents of Shai-Osudoku and Ningo-Prampram Districts, Ghana, May 2013–April 2015. BMC Infect. Dis..

[25] Shi T., McAllister D.A., O’Brien K.L., Simoes E.A.F., Madhi S.A., Gessner B.D., Polack F.P., Balsells E., Acacio S., Aguayo C. (2017). Global, regional, and national disease burden estimates of acute lower respiratory infections due to respiratory syncytial virus in young children in 2015: A systematic review and modelling study. Lancet.

[26] Kwofie T.B., Anane Y.A., Nkrumah B., Annan A., Nguah S.B., Owusu M. (2012). Respiratory viruses in children hospitalized for acute lower respiratory tract infection in Ghana. Virol. J..

[27] Wilson P.T., Baiden F., Brooks J.C., Giessler K.M., Apio G., Punguyire D., Moresky R.T., Sylverken J., Nyarko-Jectey K., Tagbor H. (2018). Respiratory Pathogens in Children 1 Month to 5 Years of Age Presenting with Undifferentiated Acute Respiratory Distress in 2 District-Level Hospitals in Ghana. J. Pediatr. Infect. Dis. Soc..

[28] Bonney J.H.K., Kronmann K.C., Lindan C.P., Asante I.A., Parbie P., Aboagye J., Amankwah J., Odoom J.K., Adjabeng M., Nzussouo N.T. (2012). Virological Surveillance of Influenza-Like Illness Among Children in Ghana, 2008–2010. J. Infect. Dis..

[29] Hogan B., Ammer L., Zimmermann M., Binger T., Krumkamp R., Sarpong N., Rettig T., Dekker D., Kreuels B., Reigl L. (2017). Burden of influenza among hospitalized febrile children in Ghana. Influenza Other Respir. Viruses.

[30] Donkor E.S., Annan J.A., Badoe E.V., Dayie N.T.K.D., Labi A.-K., Slotved H.-C. (2017). Pneumococcal carriage among HIV infected children in Accra, Ghana. BMC Infect. Dis..

[31] Dayie N.T.K.D., Tettey E.Y., Newman M.J., Bannerman E., Donkor E.S., Labi A.-K., Slotved H.-C. (2019). Pneumococcal carriage among children under five in Accra, Ghana, five years after the introduction of pneumococcal conjugate vaccine. BMC Pediatr..

[32] Duedu K.O., Gyamfi J., Ayivor-Djanie R., Afenya G., Agbuglah I.B., Agbogli H.K., Essandoh P., Kugbemanya S., Adiku T.K. (2024). Co-infections of SARS-CoV-2 with respiratory syncytial virus and human influenza A in patients with symptoms of COVID-19 in Ghana: A retrospective study. New Microbes New Infect..

[33] Asante I.A., Nyarko S.O., Awuku-Larbi Y., Obeng R.A., Sarpong G.M., Amenuvor E.A.A., Adusei-Poku M., Boatemaa L., Magnusen V., Wutsika J. (2024). Decreased influenza activity during the COVID-19 pandemic in Ghana, 2020. Front. Public Health.

[34] Durizzo K., Awoonor-Williams K., Harttgen K., Günther I. (2024). Unpacking the impact of COVID-19 on child immunization: Evidence from Ghana. BMC Public Health.

[35] Graham H.R., Bakare A.A., Ayede A.I., Gray A.Z., McPake B., Peel D., Olatinwo O., Oyewole O.B., Neal E.F.G., Nguyen C.D. (2019). Oxygen systems to improve clinical care and outcomes for children and neonates: A stepped-wedge cluster-randomised trial in Nigeria. PLoS Med..

[36] Lam F., Stegmuller A., Chou V.B., Graham H.R. (2021). Oxygen systems strengthening as an intervention to prevent childhood deaths due to pneumonia in low-resource settings: Systematic review, meta-analysis and cost-effectiveness. BMJ Glob. Health.

[37] Enweronu-Laryea C.C., Andoh H.D., Frimpong-Barfi A., Asenso-Boadi F.M. (2018). Parental costs for in-patient neonatal services for perinatal asphyxia and low birth weight in Ghana. PLoS ONE.

[38] Amegah A.K., Quansah R., Jaakkola J.J.K. (2014). Household Air Pollution from Solid Fuel Use and Risk of Adverse Pregnancy Outcomes: A Systematic Review and Meta-Analysis of the Empirical Evidence. PLoS ONE.

[39] Prah J., Kizzie-Hayford J., Walker E., Ampofo-Asiama A. (2017). Antibiotic prescription pattern in a Ghanaian primary health care facility. Pan Afr. Med. J..

[40] Opintan J.A., Newman M.J., Arhin R.E., Donkor E.S., Gyansa-Lutterodt M., Mills-Pappoe W. (2015). Laboratory-based nationwide surveillance of antimicrobial resistance in Ghana. Infect. Drug Resist..

[41] Labi A.-K., Kartey B.S., Hedidor G.K., Nuertey B.D., Kodjoe E., Vanderpuije L.N., Obeng-Nkrumah N. (2023). Antibiotic consumption trends in Ghana: Analysis of six-years pharmacy issue data from a secondary healthcare facility. JAC Antimicrob. Resist..

[42] Newman M.J., Frimpong E., Donkor E.S., Opintan J.A., Asamoah-Adu A. (2011). Resistance to antimicrobial drugs in Ghana. Infect. Drug Resist..

[43] Ofori S.K., Schwind J.S., Sullivan K.L., Cowling B.J., Chowell G., Fung I.C.-H. (2022). Transmission Dynamics of COVID-19 in Ghana and the Impact of Public Health Interventions. Am. J. Trop. Med. Hyg..

[44] WHO Essential Health Services Face Continued Disruption During COVID-19 Pandemic. https://www.who.int/news/item/07-02-2022-essential-health-services-face-continued-disruption-during-covid-19-pandemic.

[45] Graham H.R., King C., Rahman A.E., Kitutu F.E., Greenslade L., Aqeel M., Baker T., Brito L.F.d.M., Campbell H., Czischke K. (2025). Reducing global inequities in medical oxygen access: The Lancet Global Health Commission on medical oxygen security. Lancet Glob. Health.

[46] Janssen J., Afari-Asiedu S., Monnier A., Abdulai M.A., Tawiah T., Wertheim H., Baltussen R., Asante K.P. (2022). Exploring the economic impact of inappropriate antibiotic use: The case of upper respiratory tract infections in Ghana. Antimicrob. Resist. Infect. Control.

[47] Donkor E.S., Odoom A., Osman A.-H., Darkwah S., Kotey F.C.N., Donkor E.S., Odoom A., Osman A.-H., Darkwah S., Kotey F.C.N. (2024). A Systematic Review on Antimicrobial Resistance in Ghana from a One Health Perspective. Antibiotics.

[48] PATH PATH Applauds WHO Recommendation on RSV Immunization. https://www.path.org/our-impact/media-center/path-welcomes-who-global-recommendation-on-rsv-immunization/.

[49] UNICEF Ghana 6 Ways UNICEF is Supporting Immunisation in Ghana. https://www.unicef.org/ghana/stories/6-ways-unicef-supporting-immunisation-ghana%E2%80%AF.

[50] UNICEF Immunization|UNICEF Ghana. https://www.unicef.org/ghana/immunization.

[51] Fahad H. (2025). MSc Strengthening Health for the Future: Ghana’s Immunization Efforts and Strategic Plans—Vaccine Alliance. https://www.vaccinealliance.org/resources/ghana_injection_safetyfinal/.

[52] Labi A.-K., Obeng-Nkrumah N., Nartey E.T., Bjerrum S., Adu-Aryee N.A., Ofori-Adjei Y.A., Yawson A.E., Newman M.J. (2018). Antibiotic use in a tertiary healthcare facility in Ghana: A point prevalence survey. Antimicrob. Resist. Infect. Control.

[53] Ministry of Health Strategic Action Plan for the National Medical Oxygen Policy 2023–2027. https://www.moh.gov.gh/wp-content/uploads/2023/10/Strategic-Action-Plam_National-Medical-Oxygen-Policy.pdf.

[54] WHO WHO Outlines Recommendations to Protect Infants Against RSV—Respiratory Syncytial Virus. https://www.who.int/news/item/30-05-2025-who-outlines-recommendations-to-protect-infants-against-rsv-respiratory-syncytial-virus.

[55] Fradique A., Rodrigues M., Azevedo e Sousa L.I., Jorge A. (2025). Changes in Pediatric Acute Respiratory Infections Before, During, and After the COVID-19 Pandemic: A Retrospective Study. Cureus.

[56] Hesse C.A., Kpeglo E.D., Boyetey D.B., Nadarajah S. (2025). Impact of the COVID-19 pandemic on number of deaths in Ghana: An application of ARIMA with intervention analysis. Front. Public Health.

[57] Amponsah O.K.O., Buabeng K.O., Owusu-Ofori A., Ayisi-Boateng N.K., Hämeen-Anttila K., Enlund H. (2021). Point prevalence survey of antibiotic consumption across three hospitals in Ghana. JAC Antimicrob. Resist..

[58] Owusu M., Nkrumah B., Acheampong G., Opoku Afriyie S., Addae E.K., Larbi R., Ansah R.O., Kubio C., Saeed F., Ayisi-Boateng N.K. (2025). Performance of novel digital real-time PCR for detection of SARS-CoV-2, respiratory syncytial viruses, and influenza viruses in Ghana. Microbiol. Spectr..

[59] Ekholuenetale M., Nzoputam C.I., Okonji O.C., Barrow A., Wegbom A.I., Edet C.K. (2023). Differentials in the prevalence of acute respiratory infections among under-five children: An analysis of 37 sub-Saharan countries. Glob. Pediatr. Health.

